# Supplementary material for: A simple method to efficiently generate structural variation in plants
Source: PLoS Genet. 2025 Dec 18;21(12):e1011977. doi: 10.1371/journal.pgen.1011977 (PMC12725597; doi:10.1371/journal.pgen.1011977)
Supplement: S1 Text — (DOCX) [file pgen.1011977.s001.docx]

**S1 Text**

**A simple method to efficiently generate structural variation in plants**

**Supplementary Methods**

**Safety**

Etoposide is a cytotoxic drug and etoposide-containing waste should be treated in accordance with institutional guidelines. In our case, all etoposide-containing waste was incinerated. Previous studies have shown that etoposide may be degraded by potassium permanganate or bleach[1] but we have not tested this approach. All exposure to etoposide was controlled via the use of personal protective equipment. **Plant Growth Conditions**

All plants were grown in a Conviron growth chamber at 22°C with 16 h of 120 µmol light per day.

**Phenotyping**

Between 25 and 75 seeds from each M1 line were planted over three phases; M2 plants with visible phenotypes were manually identified. To identify mutants with leaf-shape differences, we measured leaf shape and size in mutant lineages where M2/M3 populations included three or more individuals displaying similar mutant phenotypes. Leaves were excised at the base of the petiole, arranged in decreasing size order on white paper, and scanned at 600 dpi on an Epson V600 scanner. Six automated leaf shape measurements were obtained (blade area, circularity, length, perimeter, width, and petiole length) using the LeafJ plugin for ImageJ software [2]. Leaf shape was manually traced in cases where leaf boundaries were not distinguished by ImageJ.

**RNA sequencing and analysis**

RNA sequencing was conducted on M3 generation plants. Six plants of each *BR-like dwarf, short-internode dwarf*, and *virescent* phenotypes, as well as four non-phenotypic individuals corresponding to sibling lines of each phenotype were sequenced. For the fraction variegated line, thirteen green plants (three of which produced ~25% variegated progeny) and one white plant were sampled. Nine M3 progeny of DMSO-treated plants were included as controls.

Mature leaf tissue from 32-day-old plants was flash frozen in liquid nitrogen. Frozen tissue was ground into a powder with a Tissuelyser II (QIAGEN; 30 beats/sec for 30 sec). RNA was extracted using an RNeasy Plant Mini Kit (QIAGEN). Library preparation was performed at the MIT BioMicro Center using an NEBNext Ultra II Directional RNA Library Prep Kit for Illumina (New England Biolabs). Samples were multiplexed on one lane of an Illumina NovaSeq 6000 S4 flow cell and sequenced at the MIT BioMicro Center with 150 bp paired end reads.

Trim Galore [3] was used to remove adapters and low-quality ends from reads (*--paired --retain_unpaired -q 25*). Salmon [4] was used to quantify the expression of transcripts, utilizing the Araport11 transcriptome as the reference and the Col-CEN v1.2 genome as decoy sequences. Five samples were identified as outliers and removed from the dataset, based upon the PCAgrid function in the R package rrcov [5] and preliminary analyses. DESeq2 [6] was used to quantify differential expression; for a gene to be considered it must have at least 10 normalized counts in four or more samples. Each phenotype of interest was analyzed independently; DESeq2 design (*~ treatment + phenotype*) incorporated both treatment (etoposide or DMSO) and phenotype (present or absent). The R package clusterProfiler [7] was used to conduct gene set enrichment analysis. Data was visualized utilizing R and packages ggplot2, EnhancedVolcano [8], and pheatmap [9].

**Identification of structural variants: short-read sequencing**

DNA was extracted from the rosette leaves of M2 and M3 plants using a CTAB protocol. Up to 15 ng of genomic DNA was used by the BioMicro Center at MIT to construct DNA sequencing libraries using the Nextera DNA Flex library preparation kit. Libraries were multiplexed and sequenced in 150 bp paired-end format using an Illumina NovaSeq 6000 S4. The first 10 bp of each read as well as any adaptor sequence was removed using Trimmomatic [10] and aligned to the Col-0 Centromere assembly [11] using BWA-MEM [12] (*bwa mem -R "@RG\tID:id\tSM:sample\tLB:lib" reference.fasta sample.R1.fastq sample.R2.fastq \ | samblaster --excludeDups --addMateTags --maxSplitCount 2 --minNonOverlap 20*). These alignments were used to identify structural variants by two separate methods. Larger deletions and duplications were identified using a method proposed by the Comai Lab [13]. Coverage was calculated for each 100 kb window for properly paired and aligned reads using Bedtools [14] (*bedtools coverage -a 100kb_windows.bed -b sample_paired_sorted.bam -counts*) and read-counts/window for each line was normalized to library size as reads per million. The median normalized read-counts for all lines for each window was calculated. As most lines are not expected to share a structural variant, this median read depth is considered to be the wild-type genome dosage and is set to a ploidy or chromosomal dosage of two. The chromosomal dosage of any window for a given line is then calculated as (*normalized read-counts / median read-counts) x2.* Windows with a coverage score ≥3 were considered heterozygous duplications, ≥4 to be homozygous duplications, ≤1 to be heterozygous deletion and ≤0 to be a homozygous deletion.
In addition, we also identified structural variation using a benchmarked Lumpy Express pipeline [15] . Briefly, Samtools [16] was used to identify discordant and split reads from the SAM file that was output by BWA . Files containing discordant reads, split reads, and all reads were used by Lumpy Express to identify putative structural variants. Due to mapping artifacts, Lumpy Express and other variant callers often identify false-positive structural variant candidates at repetitive regions. To address this problem, we filtered out variants from ChrC, ChrM, and the NOR on chromosome 2. To remove SVs present in lab wild-type Col-0 stocks, we removed SVs within 2 kb of any SV found in lines only treated with DMSO, or SVs shared by four or more unrelated etoposide-treated mutant lineages. Finally, only those deletions and duplications that were supported by split-reads were retained in the final list of SVs.

To assess if etoposide causes single nucleotide variants, we used BWA-MEM to align reads from 32 M2/M3 progeny of etoposide-treated plants and four M2 progeny of control plants treated with DMSO to the Col-0 centromere assembly. Aligned reads were sorted and converted to variant calling format (*bcftools mpileup -O b -o file.bcf -f ref.fasta file.sorted.bam*) followed by SNP/indel calling (*bcftools call --ploidy 2 -m -v -o file.vcf file.bcf*) [16]. Variants that were called by bcftools were filtered to retain only calls with a quality score higher than or equal to 40 (*bcftools view -O z -o filtered.vcf.gz -e 'QUAL<=40' in.vcf.gz*). Variants shared by all untreated lines were extracted (*bcftools isec -n=5 -p common_positions.vcf input_files_filtered.vcf.gz*), and then excluded from all other treated lines (*bcftools view -T common_positions.vcf filtered_files.vcf -O z -o untreated_filtered_files.vcf*).

**Identification of structural variants: long-read sequencing**

Two mature leaves from 32-day-old M3 plants were flash frozen in liquid nitrogen. Frozen tissue was ground into a powder with a Tissuelyser II (QIAGEN; 30 beats/sec for 1 min). High molecular weight DNA was extracted using a Wizard HMW DNA Extraction Kit (Promega). DNA was sheared by passing through a 23G needle twice. Contaminating RNA and proteins were removed using RNase A (New England Biolabs) and Proteinase K (New England Biolabs). DNA was further cleaned and small fragments removed using a modified SPRI bead mixture in which the buffer of Agencourt AMPure XP beads was exchanged with a custom buffer of 10 mM Tris-HCl, 1 mM EDTA pH 8, 1.6 M NaCl, 11% w/v PEG 8000 [17]. To perform size selection, in brief, 0.7x volume of modified bead solution was added to each DNA sample, then incubated on a nutator for 10 min. Samples were placed on a magnetic stand to bind the beads to the side of the tube, then washed twice with 70% ethanol. Beads were not allowed to dry before adding nuclease-free water to elute the DNA. To increase yield and remove residual ethanol, samples were incubated at 50°C for 2 min and then at room temperature for two hours with the lids open.

All library preparation and sequencing was performed at the MIT BioMicro Center. For two samples showing the *BR-like dwarf* phenotype, high molecular weight DNA extractions had low yield. For these samples and corresponding controls (individual from sibling line without dwarf phenotype and individual from a line treated with DMSO), library preparation was performed utilizing a Rapid Barcoding Kit V10 (Oxford Nanopore Technologies; SQK-RBK110-96). These four samples were multiplexed and sequenced on a single PromethION R9.4.1 flow cell (Oxford Nanopore Technologies; FLO-PRO002). For all other samples, library preparation was performed with a Native Barcoding Kit V14 (Oxford Nanopore Technologies; SQK-NBD114-24). These samples were multiplexed into groups of three and each group was sequenced on its own PromethION R10.4.1 flow cell (Oxford Nanopore Technologies; FLO-PRO114M). Both runs utilized the following software versions for sequencing and base-calling: MinKNOW 23.04.6, Bream 7.5.10, Configuration 5.5.14, Guppy 6.5.7, and MinKNOW Core 5.5.5.

Nanopore reads were filtered utilizing Filtlong v0.2.1 [18] to select the best reads in terms of length and quality, up to a max coverage of 50x (*--min_length 1000 --min_window_q 40 --trim --split 1500 --target_bases 6605000000*). Filtered reads were mapped to the Col-CEN v1.2 genome using Vulcan dual-mode alignment pipeline [19], which leverages both minimap2 [20] and NGMLR [21], using default settings for ONT reads. Structural variants in each sample were detected with cuteSV *(--max_cluster_bias_INS 100 --diff_ratio_merging_INS 0.3 --max_cluster_bias_DEL 100 --diff_ratio_merging_DEL 0.3 --min_size 30 --max_size -1 –genotype;* [22,23]). SVs identified in all samples were merged with SURVIVOR (*merge 1000 1 1 1 0 30;* [24] ), then samples were genotyped with cuteSV for all SVs identified (*--min_mapq 20 --max_cluster_bias_INS 100 --diff_ratio_merging_INS 0.3 --max_cluster_bias_DEL 100 --diff_ratio_merging_DEL 0.3 --min_size 30 --max_size -1*). Structural variants present in the DMSO and/or Col-0 controls, or present in more than one phenotypic line, or with a quality less than 20 were removed. Data was visualized in R using ggplot2.

**References**

1. Benvenuto JA, Connor TH, Monteith DK, Laidlaw JL, Adams SC, Matney TS, et al. Degradation and inactivation of antitumor drugs. J Pharm Sci. 1993;82: 988–991.

2. Maloof JN, Nozue K, Mumbach MR, Palmer CM. LeafJ: An ImageJ Plugin for Semi-automated Leaf Shape Measurement. JoVE. 2013; 50028. doi:10.3791/50028

3. Krueger F. FelixKrueger/TrimGalore. 2024. Available: https://github.com/FelixKrueger/TrimGalore

4. Patro R, Duggal G, Love MI, Irizarry RA, Kingsford C. Salmon provides fast and bias-aware quantification of transcript expression. Nat Methods. 2017;14: 417–419. doi:10.1038/nmeth.4197

5. Chen X, Zhang B, Wang T, Bonni A, Zhao G. Robust principal component analysis for accurate outlier sample detection in RNA-Seq data. BMC Bioinformatics. 2020;21: 269. doi:10.1186/s12859-020-03608-0

6. Love MI, Huber W, Anders S. Moderated estimation of fold change and dispersion for RNA-seq data with DESeq2. Genome Biol. 2014;15: 550. doi:10.1186/s13059-014-0550-8

7. Wu T, Hu E, Xu S, Chen M, Guo P, Dai Z, et al. clusterProfiler 4.0: A universal enrichment tool for interpreting omics data. Innovation (Camb). 2021;2: 100141. doi:10.1016/j.xinn.2021.100141

8. Blighe K, Rana S, Lewis M. EnhancedVolcano: Publication-ready volcano plots with enhanced colouring and labeling. 2018. Available: https://github.com/kevinblighe/EnhancedVolcano

9. Kolde R. pheatmap: Pretty Heatmaps. 2019. Available: https://cran.r-project.org/web/packages/pheatmap/index.html

10. Bolger AM, Lohse M, Usadel B. Trimmomatic: a flexible trimmer for Illumina sequence data. Bioinformatics. 2014;30: 2114–2120. doi:10.1093/bioinformatics/btu170

11. Naish M, Alonge M, Wlodzimierz P, Tock AJ, Abramson BW, Schmücker A, et al. The genetic and epigenetic landscape of the Arabidopsis centromeres. Science. 2021;374: eabi7489. doi:10.1126/science.abi7489

12. Li H. Aligning sequence reads, clone sequences and assembly contigs with BWA-MEM. arXiv; 2013. doi:10.48550/arXiv.1303.3997

13. Tan E, Comai L, Henry I. Chromosome Dosage Analysis in Plants Using Whole Genome Sequencing. BIO-PROTOCOL. 2016;6. doi:10.21769/BioProtoc.1854

14. Quinlan AR, Hall IM. BEDTools: a flexible suite of utilities for comparing genomic features. Bioinformatics. 2010;26: 841–842. doi:10.1093/bioinformatics/btq033

15. Göktay M, Fulgione A, Hancock AM. A New Catalog of Structural Variants in 1,301 A. thaliana Lines from Africa, Eurasia, and North America Reveals a Signature of Balancing Selection at Defense Response Genes. Molecular Biology and Evolution. 2021;38: 1498–1511. doi:10.1093/molbev/msaa309

16. Danecek P, Bonfield JK, Liddle J, Marshall J, Ohan V, Pollard MO, et al. Twelve years of SAMtools and BCFtools. GigaScience. 2021;10: giab008. doi:10.1093/gigascience/giab008

17. Schalamun M, Nagar R, Kainer D, Beavan E, Eccles D, Rathjen JP, et al. Harnessing the MinION: An example of how to establish long-read sequencing in a laboratory using challenging plant tissue from Eucalyptus pauciflora. Molecular Ecology Resources. 2019;19: 77–89. doi:10.1111/1755-0998.12938

18. Wick R. rrwick/Filtlong. 2024. Available: https://github.com/rrwick/Filtlong

19. Fu Y, Mahmoud M, Muraliraman VV, Sedlazeck FJ, Treangen TJ. Vulcan: Improved long-read mapping and structural variant calling via dual-mode alignment. Gigascience. 2021; 10: giab063. doi: 10.1093/gigascience/giab063

20. Li H. Minimap2: pairwise alignment for nucleotide sequences. Bioinformatics. 2018;34: 3094–3100. doi:10.1093/bioinformatics/bty191

21. Sedlazeck FJ, Rescheneder P, Smolka M, Fang H, Nattestad M, von Haeseler A, et al. Accurate detection of complex structural variations using single-molecule sequencing. Nat Methods. 2018;15: 461–468. doi:10.1038/s41592-018-0001-7

22. Jiang T, Liu Y, Jiang Y, Li J, Gao Y, Cui Z, et al. Long-read-based human genomic structural variation detection with cuteSV. Genome Biology. 2020;21: 189. doi:10.1186/s13059-020-02107-y

23. Jiang T, Cao S, Liu Y, Zhang Z, Liu S, Liu B, Wang G, et al. cuteFC: regenotyping structural variants through an accurate and efficient force-calling method. Genome Biology. 2025;26: 166. doi: 10.1186/s13059-025-03642-2

24. Jeffares DC, Jolly C, Hoti M, Speed D, Shaw L, Rallis C, et al. Transient structural variations have strong effects on quantitative traits and reproductive isolation in fission yeast. Nat Commun. 2017;8: 14061. doi:10.1038/ncomms14061
