# Supplementary material for: A simple method to efficiently generate structural variation in plants
Source: PLoS Genet. 2025 Dec 18;21(12):e1011977. doi: 10.1371/journal.pgen.1011977 (PMC12725597; doi:10.1371/journal.pgen.1011977)
Supplement: S1 Fig — (PDF) [file pgen.1011977.s002.pdf]

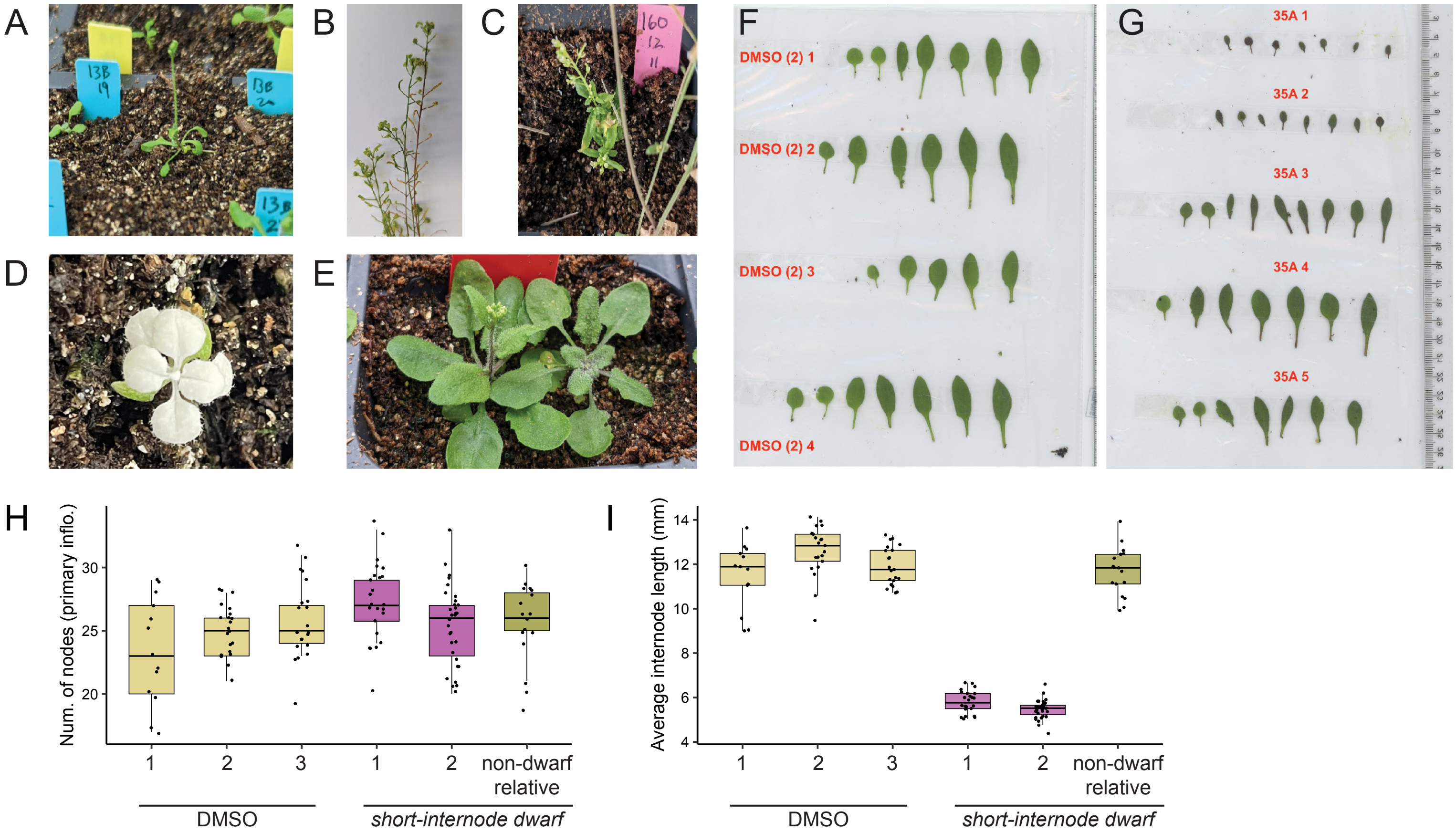

S1 Fig. Additional phenotyping of progeny of etoposide-treated plants. Visual observation of M2 plants arising from different etoposide-treated M1 progenitors identified multiple lines with mutant phenotypes, including: early flowering **(A)**, sterility **(B)**, dwarfism **(C)**, variegation **(D)**, altered leaf shape **(E-G) .** Lines with altered leaf shape or size were visually identified. These differences were confirmed by measuring leaf size (blade area, length, perimeter, width, and petiole length) and shape (cirularity) of leaves with the LeafJ plugin for ImageJ software. Leaves from multiple M2 siblings of line 35 are shown in **(G)**. 35A_1 and 35A_2 show a dramatic decrease in size parameters while 35A_3 shows an intermediate decrease. 35A_4 and 5 show no change compared to DMSO-treated lineages. **(H)** *short-internode dwarf* plants have the same number of nodes as DMSO-treated lineages and a genetic relative that lacks the phenotype, but **(I)** decreased distance between nodes, as measured on the primary inflorescence.
