# Supplementary material for: A simple method to efficiently generate structural variation in plants
Source: PLoS Genet. 2025 Dec 18;21(12):e1011977. doi: 10.1371/journal.pgen.1011977 (PMC12725597; doi:10.1371/journal.pgen.1011977)
Supplement: S2 Fig — (PDF) [file pgen.1011977.s003.pdf]

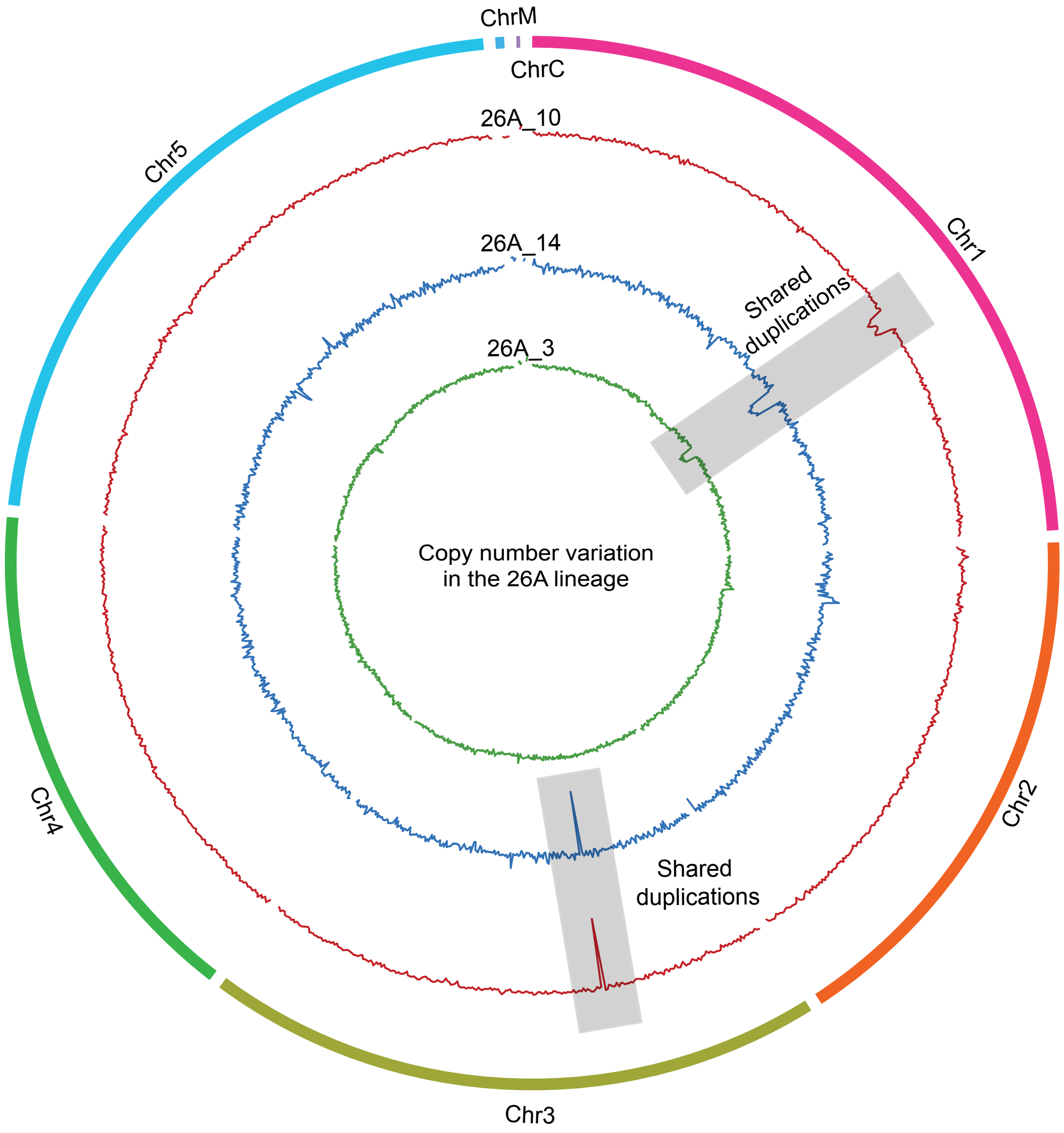

S2 Fig. Duplications detected in the 26A lineage. Each track of the circos plot represents genome-wide read coverage analysis for an M2 sibling generated by selfing the 26A M1 mutant. Chromosome 1 duplications are shared by three M2 siblings whereas a chromosome 3 duplication is shared by two of three siblings.
