# Supplementary material for: A simple method to efficiently generate structural variation in plants
Source: PLoS Genet. 2025 Dec 18;21(12):e1011977. doi: 10.1371/journal.pgen.1011977 (PMC12725597; doi:10.1371/journal.pgen.1011977)
Supplement: S3 Fig — (PDF) [file pgen.1011977.s004.pdf]

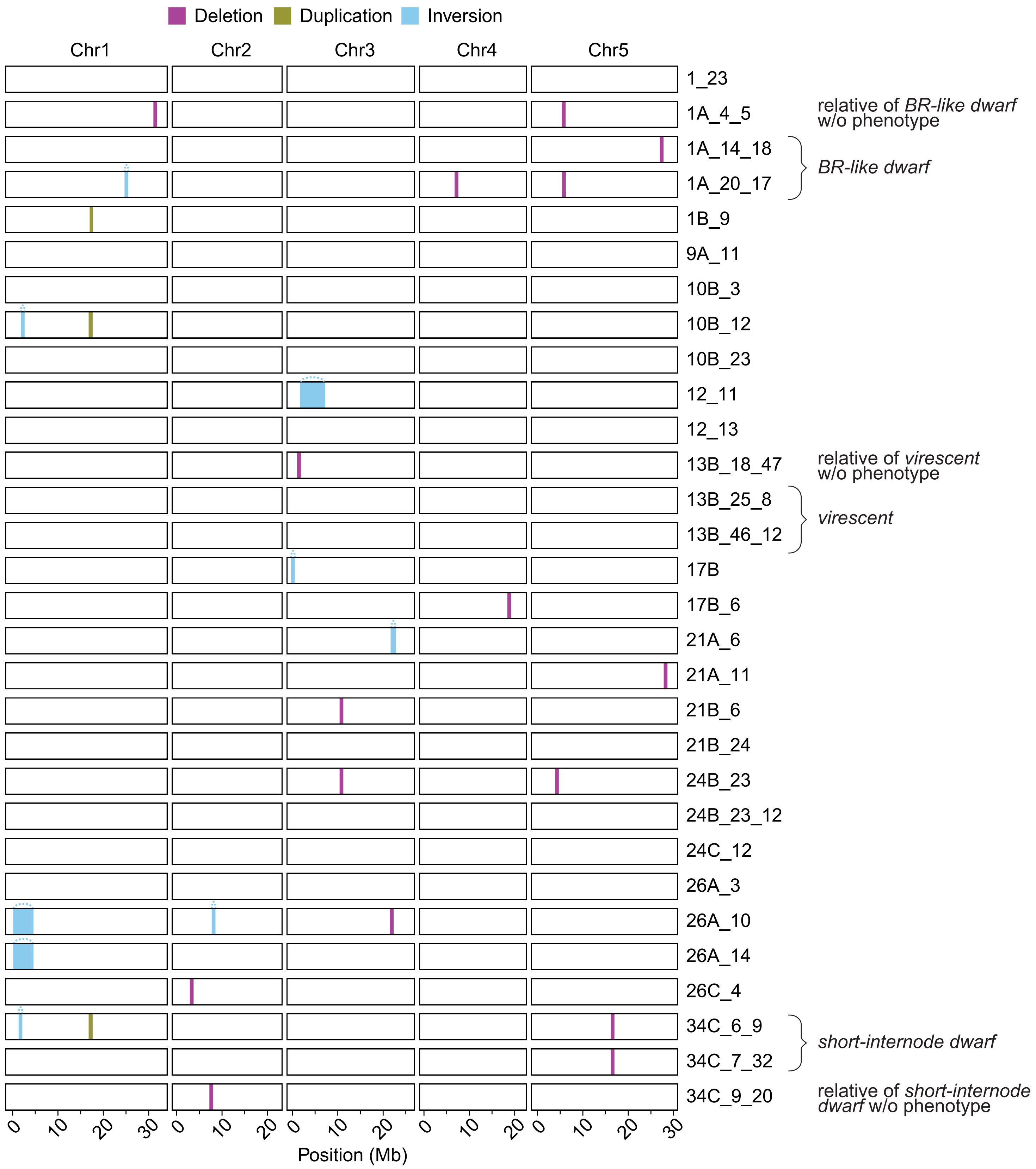

S3 Fig. Structural variants identified by LUMPY Express in short-read data. A total of 27 SVs (16 deletions, 3 duplications, and 8 inversions) were identified. Eleven sequenced samples did not have any detected SVs.
