# Supplementary material for: A simple method to efficiently generate structural variation in plants
Source: PLoS Genet. 2025 Dec 18;21(12):e1011977. doi: 10.1371/journal.pgen.1011977 (PMC12725597; doi:10.1371/journal.pgen.1011977)
Supplement: S4 Fig — (PDF) [file pgen.1011977.s005.pdf]

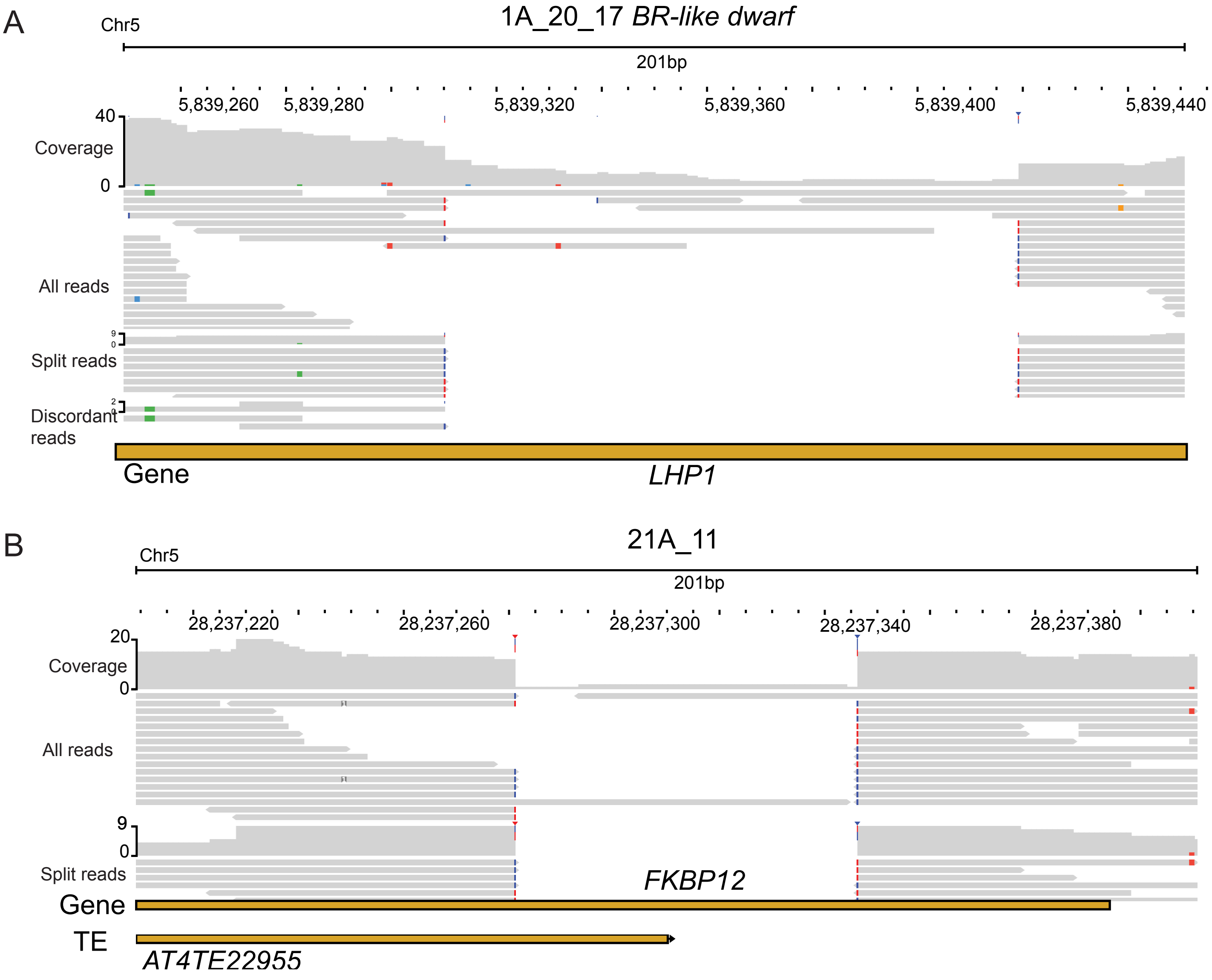


**S4 Fig. Examples of deletions in an etoposide-treated line identified by LUMPY Express using short reads.** JBrowse snapshots of split reads and all reads overlapping *LHP1* **(A)** and *FKBP12* **(B)** identifies short deletions in two independent lines.
