# Supplementary material for: A simple method to efficiently generate structural variation in plants
Source: PLoS Genet. 2025 Dec 18;21(12):e1011977. doi: 10.1371/journal.pgen.1011977 (PMC12725597; doi:10.1371/journal.pgen.1011977)
Supplement: S5 Fig — (PDF) [file pgen.1011977.s006.pdf]

Line 12_11

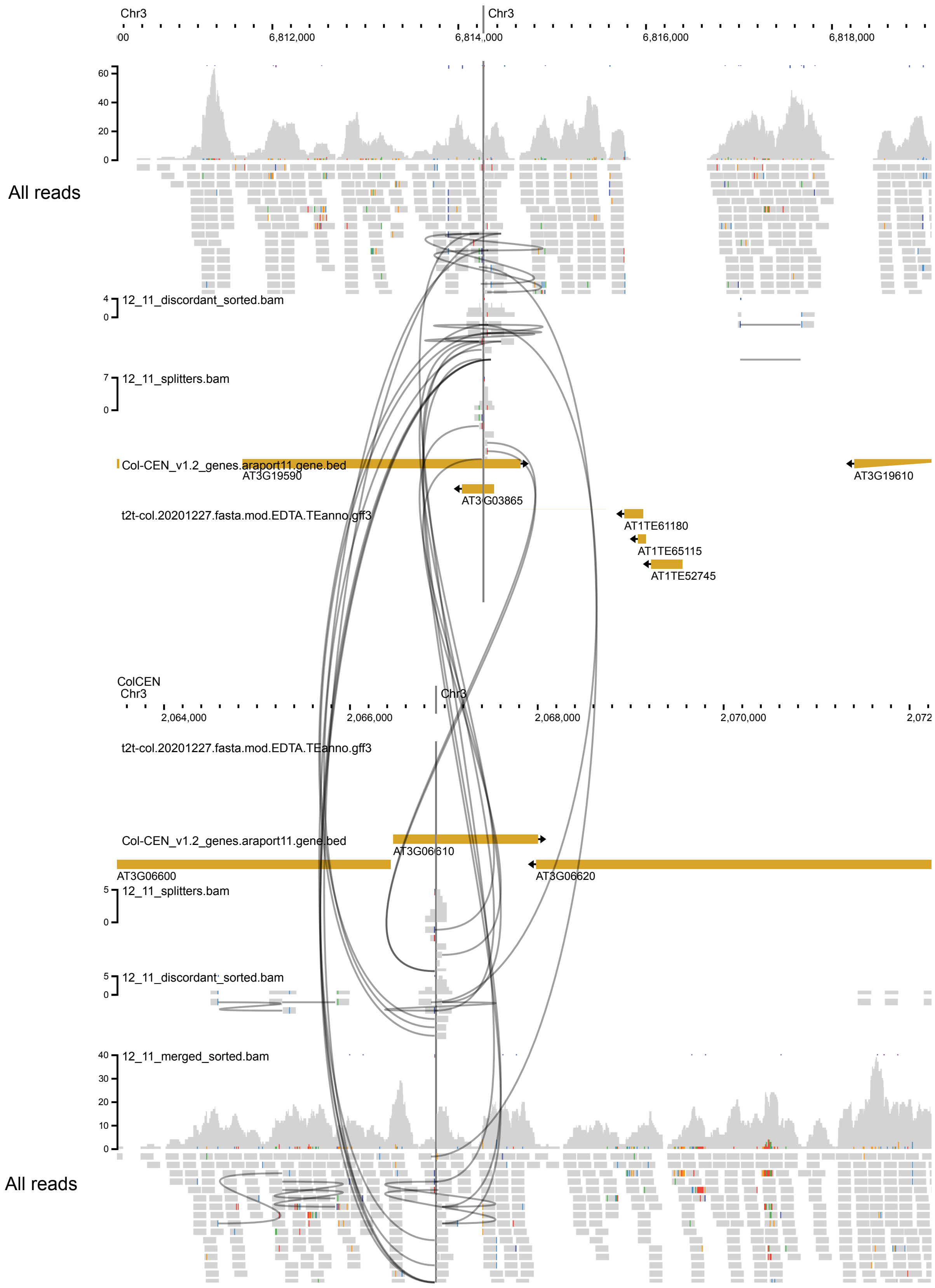

S5 Fig. An example of an intrachromosomal inversion in an etoposide-treated line detected by LUMPY Express using short reads. Split reads and discordant reads indicate an intra-chromosomal inversion on chromosome 3. Black lines link split and discordant reads mapping to two different regions of the chromosome.
