# Supplementary material for: A simple method to efficiently generate structural variation in plants
Source: PLoS Genet. 2025 Dec 18;21(12):e1011977. doi: 10.1371/journal.pgen.1011977 (PMC12725597; doi:10.1371/journal.pgen.1011977)
Supplement: S6 Fig — (PDF) [file pgen.1011977.s007.pdf]

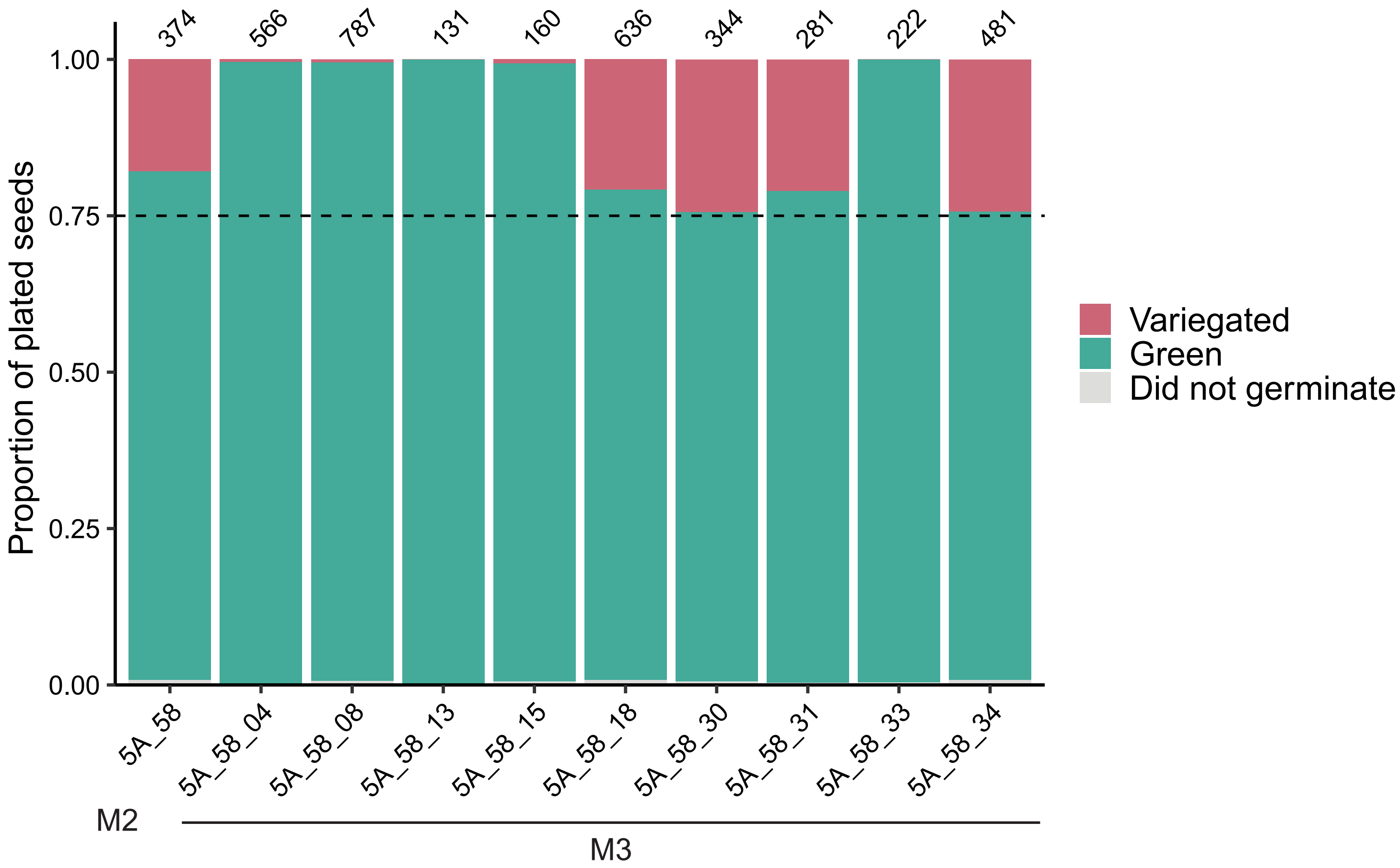

S6 Fig. Segregation of the *variegated* phenotype from green parents. Green plants from variegated line 5A produce either approximately 25% variegated progeny or all green progeny. Number of seedlings assesed is annotated above each bar. M2 plant 5A_58 is the parent of the M3 progeny assayed.
