# Supplementary material for: A simple method to efficiently generate structural variation in plants
Source: PLoS Genet. 2025 Dec 18;21(12):e1011977. doi: 10.1371/journal.pgen.1011977 (PMC12725597; doi:10.1371/journal.pgen.1011977)
Supplement: S7 Fig — (PDF) [file pgen.1011977.s008.pdf]

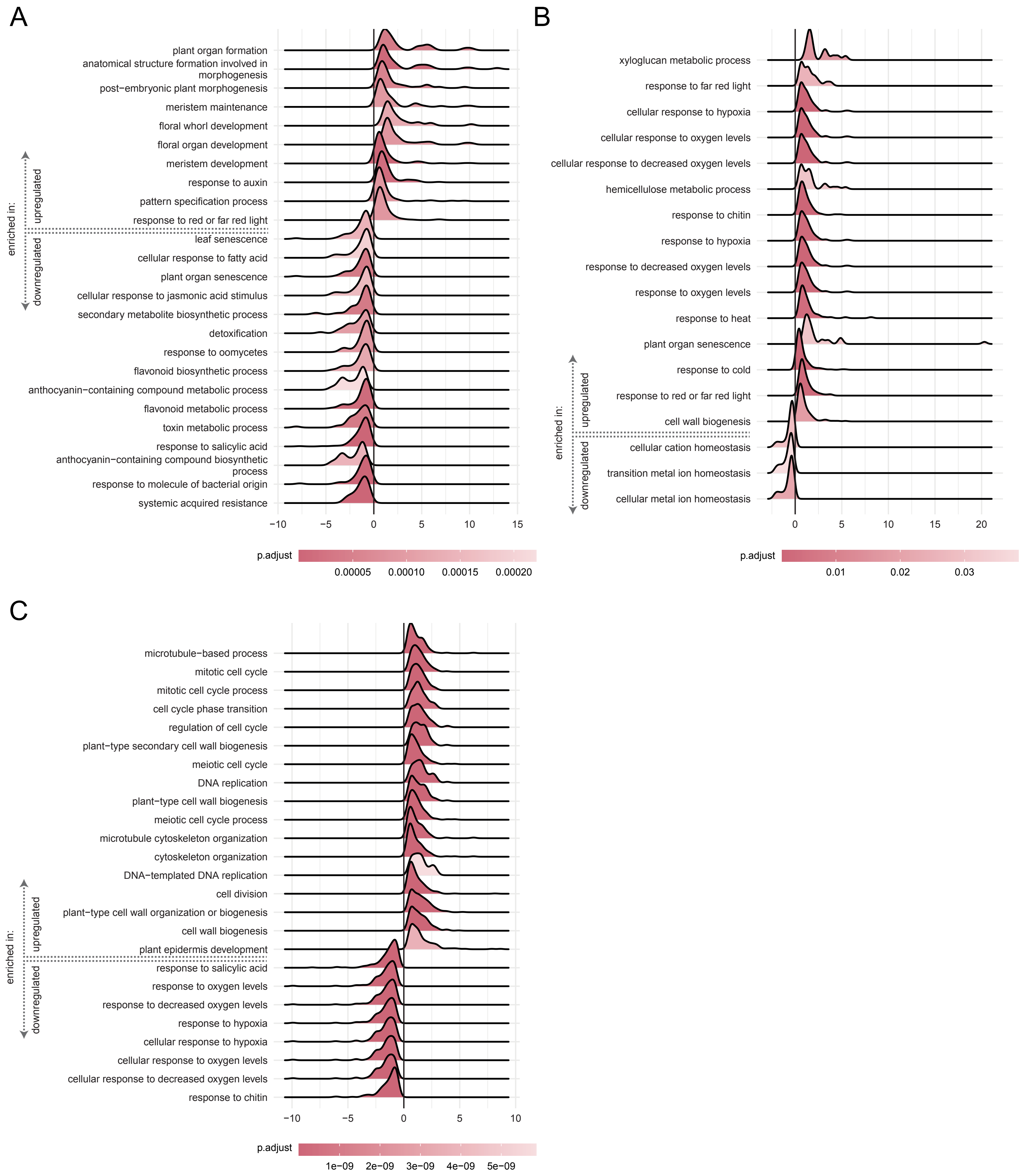

S7 Fig. GO term enrichment for genes differentially expressed between plants exhibiting a phenotype and those without a phenotype. GSEA was used to identify enriched GO terms. Ridge plots depict significant terms for **(A)** *BR-like dwarf*, **(B)** *short-internode dwarf*, and **(C)** *virescent* phenotypes.
