# Supplementary material for: A simple method to efficiently generate structural variation in plants
Source: PLoS Genet. 2025 Dec 18;21(12):e1011977. doi: 10.1371/journal.pgen.1011977 (PMC12725597; doi:10.1371/journal.pgen.1011977)
Supplement: S8 Fig — (PDF) [file pgen.1011977.s009.pdf]

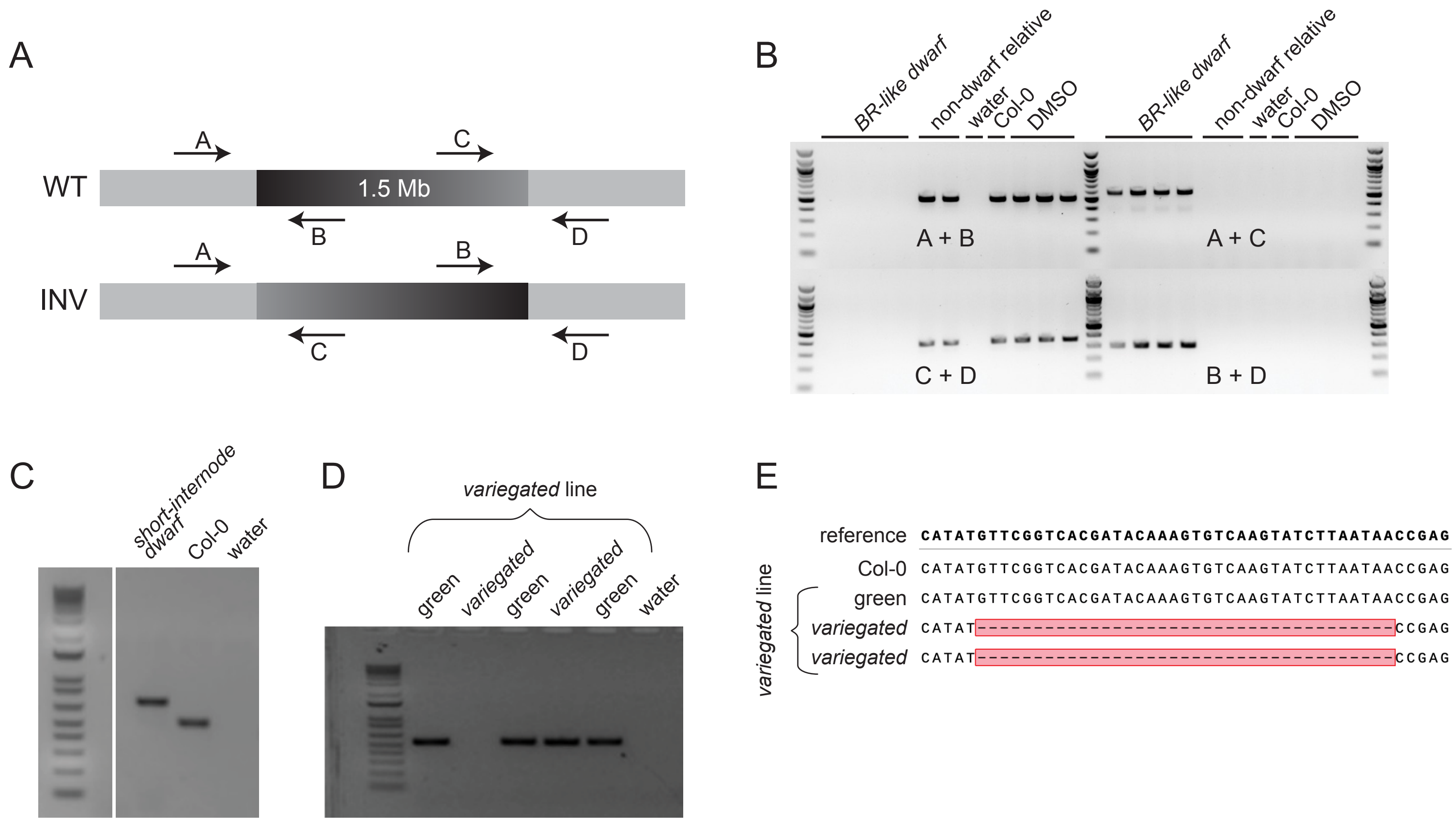

S8 Fig. PCR verification of four structural variants identified via long-read sequencing. All plants sampled for PCR verification were relatives of the plants sequenced. **(A)** Diagram depicting primer design for verifcation of 1.52 Mb inversion identified in *BR-like dwarf* plants. **(B)** Image of gel electrophoresis of PCR products generated with different combinations of primers in (A); the inversion is present only in plants exhibiting the *BR-like dwarf* phenotype. **(C)** Gel image for verification of 184 bp insertion identified in *short-internode dwarf* plants. Primers were designed to flank the insertion. **(D)** Gel image for verification of 81 bp deletion identified in the plant sequenced from the *variegated* line. Primers were designed to flank the deletion; all plants tested are homozygous for the deletion, except for one plant (lane 3) for which the PCR failed. **(E)** Alignment of sequenced PCR products to *IMMUTANS* reference sequence. Primers were designed to flank the deletion. Individuals in the line exhibiting the *variegated* phenotype are homozygous for the deletion while green plants are either heterozygous or homozygous for the WT allele.
