# Supplementary material for: A simple method to efficiently generate structural variation in plants
Source: PLoS Genet. 2025 Dec 18;21(12):e1011977. doi: 10.1371/journal.pgen.1011977 (PMC12725597; doi:10.1371/journal.pgen.1011977)
Supplement: S9 Fig — (PDF) [file pgen.1011977.s010.pdf]

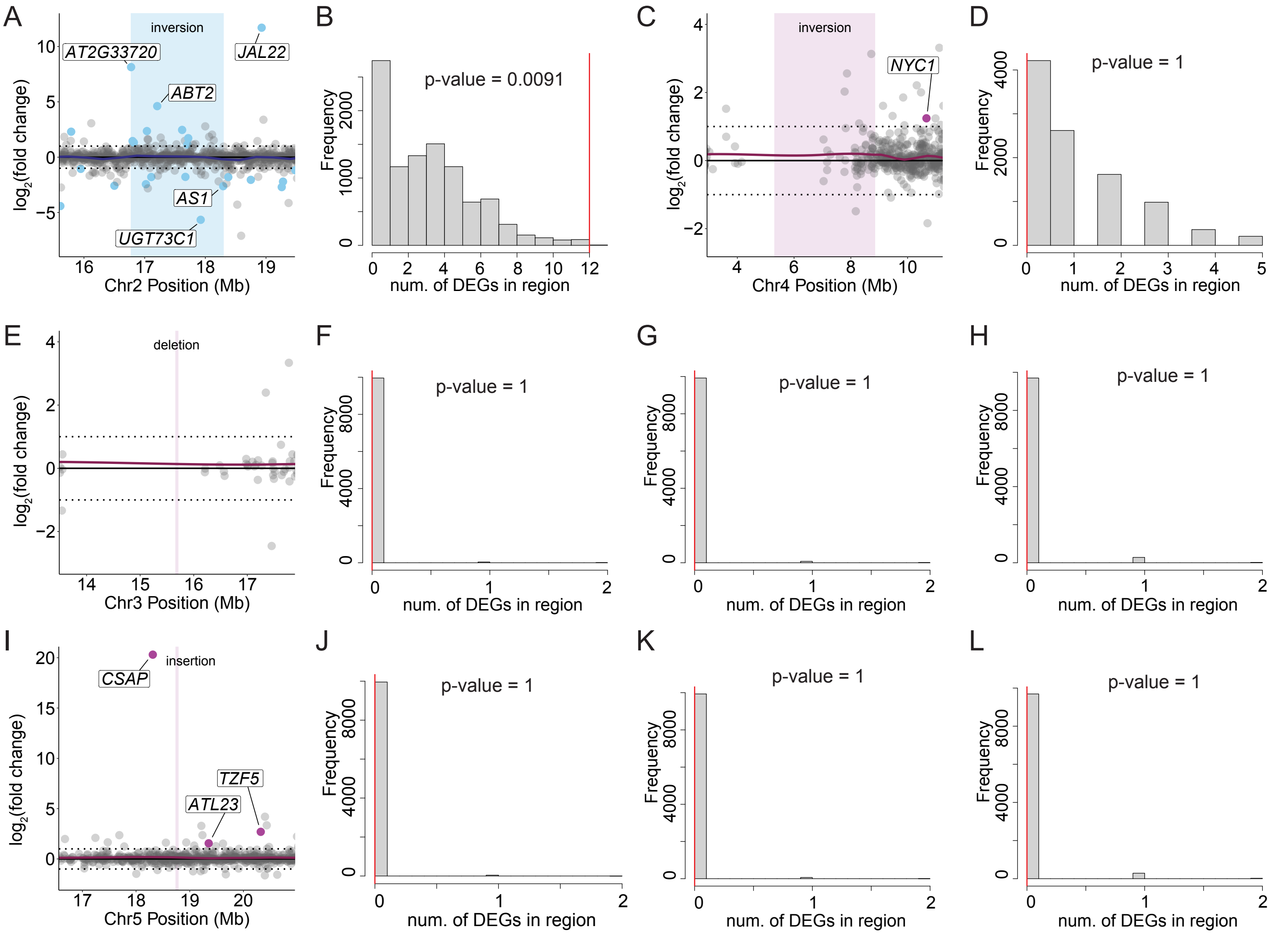

S9 Fig. Permutation tests to determine if structural variants are associated with the location of differentially expressed genes. (A) Log_2(fold change) of genes within and 1 Mb outside the 1.52 Mb inversion in *BR-like dwarf.* Differentially expressed genes (DEGs) are in blue and SVs are shaded in light blue. Dark blue line represents locally estimated scatterplot smoothing to aid pattern visualization. **(B)** Permutation test to determine if the 1.52 Mb inversion in *BR-like dwarf* plants contains more DEGs than expected by chance. All permutation tests were performed by selecting at random 10,000 genomic regions of an equivalent size to the region of interest. Histogram plots the distribution of number of DEGs contained within the randomly selected regions. Red line marks the number of DEGs in the region of interest. P-values calculated by the probability that permuted values exceed the observed value; if p-value is < 0.05 we conclude the region of interest is enriched for DEGs. **(C)** Log_2(fold change) of genes within and 2 Mb outside the 3.54 Mb inversion in *short-internode dwarf.* DEGs are in pink and SVs are shaded in light pink. Dark pink line represents locally estimated scatterplot smoothing to aid pattern visualization. **(D)** Permutation test for 3.54 Mb inversion in *short-internode dwarf* plants. **(E)** Log_2(fold change) of genes 2 Mb outside 176 bp deletion in *short-internode dwarf.* DEGs are in pink and SVs are shaded in light pink. Dark pink line represents locally estimated scatterplot smoothing to aid pattern visualization. Permutation tests for **(F)** 5 kb, **(G)** 10 kb, and **(H)** 50 kb flanking the deletion on chromosome 3 in *short-internode dwarf* plants. **(I)** Log_2(fold change) of genes 2 Mb outside 184 bp insertion in *short-internode dwarf.* DEGs are in pink and SVs are shaded in light pink. Dark pink line represents locally estimated scatterplot smoothing to aid pattern visualization. Permutation tests for **(J)** 5 kb, **(K)** 10 kb, and **(L)** 50 kb flanking the insertion on chromosome 5 in *short-internode dwarf* plants.
