# Supplementary material for: A simple method to efficiently generate structural variation in plants
Source: PLoS Genet. 2025 Dec 18;21(12):e1011977. doi: 10.1371/journal.pgen.1011977 (PMC12725597; doi:10.1371/journal.pgen.1011977)
Supplement: Attachment — Submitted filename: 153827_1_rebuttal_3383388_sw8ht4.docx [file pgen.1011977.s022.docx]

**Manuscript number:** RC-2024-02840

**Corresponding author(s):** PRV Satyaki & Mary Gehring

## 1. General Statements [optional]

*This section is optional. Insert here any general statements you wish to make about the goal of the study or about the reviews.*

## 2. Point-by-point description of the revisions

*This section is mandatory.* *Please insert a point-by-point reply describing the revisions that were already carried out and included in the transferred manuscript.*

**Reviewer 1**

This is review of the manuscript „A simple method to efficiently generate structural variation in plants" by Bechen et al. The manuscript presents a very interesting and innovative approach to generate structural variant mutations (including large ones) in the genome of Arabidopsis thaliana using a simple chemical treatment with TOPII inhibitor etoposide. Authors show that unlike chemical mutagens commonly used for induction of SNPs (EMS, sodium azide...), etoposide-treatment caused structural variants like DNA deletions, insertions, inversions and translocations. These mutations were identified by the whole genome short and long read sequencing that also indicated a WT-like frequency of SNPs. This finding can potentially help inducing mutations similar to high energy radiation in potentially any plant. First, the manuscript provides description of the unusual phenotypes found after etoposide treatment and their Mendelistic inheritance. Based on this, authors performed whole genome sequencing and mutation detection, validation. The experimental part ends by transcriptome analysis that authors use as the approach to identify the causal mutations. This part is, in my opinion, the weakest part of the manuscript and would benefit from further clarification or even additional experiments (see below). Overall the manuscript is very clear and contains all necessary information. The only part that was confusing to me, was the section focusing on the transcriptome analysis.

**Response:** Thank you for your appreciation of the study. As detailed below, we have changed our presentation of the RNA-seq results to better describe their purpose.

Major points: 
Line 222: In the section „RNA-Seq identifies genes that are associated with structural variation and mutant phenotypes", authors suggest that the changes in the transcript amount were used to identify causal mutations. I got confused by this section. Exach of the examples represents unique situation and thus only single cases are presented which makes it hard to estimate robustness of the presented approach. Also, the presented mutations have prominent phenotypes that were already heavily studied in the past and therefore the possible causal genes are mostly known. Therefore, I am not sure how this approach would stand in case of traits with unknown underlying genes.

**Response:** Our intent was not to present a new method for mapping causative mutations. Like any other induced genetic mutation, there are many possible strategies for identifying the causative locus (loci), such as mapping-by-sequencing via a segregating F2 population (as mentioned below). Organization of the manuscript’s results has been changed to reflect that mRNA-seq was conducted to learn more about the phenotypes, not to definitively identify causal genes/mutations. We have also added additional text to the discussion to clarify candidate mutation mapping approaches on line 376: “How do we identify genetic changes that are causative for phenotypes of interest? Future studies will accelerate candidate-gene discovery by employing structural-variant callers, *de novo* genome assembly-based approaches, and RNA-Seq based mapping (Mahmoud et al 2019). Although our study did not aim to use RNA-Seq to identify mutations, it provides an example of how RNA-Seq data in tandem with genome sequencing can help shortlist potential causal mutations. In cases where a potential causative variant is not obvious, these strategies can be combined with traditional genetic mapping approaches. However, mapping-by-sequencing approaches might not be easily applicable to some classes of mutants. For example, inversions or translocations can suppress recombination and reduce the efficacy of mapping-by-sequencing.”

When refering to the case with the chromosomal inversion, I do not see how one will be able to map a candidate based on the relatively mild expression (but maybe I am missing something here). Similarly, the „mapping" approach applied to the variegated line would not be possible on a trait that is less studied and the candidates are not well known. I wonder why authors did not perform association mapping on a bulk of phenotypically mutant plants collected from a segregating F2 backcross population. This might be a more robust way of linking the phenotype with a mutation.

**Response:** Our primary goal with the manuscript was to demonstrate that etoposide-treatment induces mutant phenotypes and structural variation. Identifying the causal mutation for every phenotype is outside the scope of the present study. As described above, we have added additional text in the discussion to briefly describe candidate mutation mapping approaches that researchers can use.

Discussion section: I am missing discussion on how etoposide could be causing such structural variants.

**Response:** Etoposide’s mode of action is well-studied in animal systems and has been described in text that has been moved from results to introduction. Starting on line 90 it reads “Topo II relaxes torsional stress from DNA supercoiling generated during DNA replication or transcription by transiently breaking both strands and then ligating them after passing a DNA segment through the break. Between strand breakage and ligation, Topo II is covalently linked to DNA via a tyrosine residue, forming a topoisomerase cleavage complex [37]. This complex is stabilized by the inhibitor etoposide. A collision between covalently-linked Topo II and DNA polymerases during DNA replication, or with RNA polymerases during transcription, leads to removal of the Topo II enzyme, which results in the generation of double-stranded breaks (DSBs) [38–41]. The imprecise repair of DSBs leads to genomic rearrangements and structural variation in mouse spermatocytes, fibroblasts, and in human cells [42–44]. Previously, it was shown that treatment with etoposide inhibits plant growth[45,46] and causes fragmentation of chromosomes during meiosis in Arabidopsis [45]. However, its potential as a mutagen that can induce structural variation has not been investigated"

Minor points:

Line 70: Possibly add sodium azide. It is frequently used as mutagen for some plant species.

**Response:** Added sodium azide to line 76.

Line 122: „...etoposide is an excellent mutagen for efficiently creating large-effect mutations." This cannot be claimed at this point because the sequence analysis data were not shown yet. Please reformulate.

**Response:** We changed this line (now line 133) to: “The large proportion of plants showing visible phenotypes suggested that etoposide could be an excellent mutagen for efficiently creating large-effect mutations.”

**Referees cross-commenting** 
My main issue was the mapping protocol using transcriptomic changes. It is hard to believe that this approach would work well on unknown/less studied traits. What is your opinion?

**Response:** Identifying the causal mutation for every phenotype is outside the scope of the present study. Organization of the manuscript’s results has been changed to reflect that RNA-seq was conducted to learn more about the phenotypes, not to definitively identify causal genes/mutations. In some instances (*BR-like dwarf*), the RNA-seq data, combined with prior knowledge, suggested a causal variant (*AS1*), which was further bolstered by the identification of structural variants. Note that it is only for the *variegated* mutant that we definitively identified the causal mutation (*IM*) by genetic complementation.

Reviewer #1 (Significance (Required)):

Strengths - innovative way on how to induce structural variant mutations in plants.

Limitations - The approach on how to map the mutations needs more development. At this point i tis not clear how well the approach will work in other plant species.

Audience - basic and applied plant scientists

**Response**: We have adjusted our discussion of the role of mRNA-seq in the study, and added comments on approaches for mapping causative mutations. We hope this now clarifies the overall strategy. Topoisomerase’s sensitivity to etoposide inhibition is conserved amongst tested plants and animal species. We have changed the sentence and added references to introduction to show that etoposide acts on other plant species (line 100): “Previously, it was shown that treatment with etoposide impacts genome stability and inhibits plant growth in *Arabidopsis thaliana*, *Allium cepa*, and *Lathyrus sativus* [46-49] and causes fragmentation of chromosomes during meiosis in Arabidopsis [48]. “ In addition, preliminary (unpublished) work in our labs shows that etoposide has mutagenic impacts on legumes and *Brassicaceae* crop species. We therefore believe that this protocol should be widely applicable to other plant species.

**Reviewer 2**

- The manuscript describes mutagenesis of Arabidopsis by a topoisomerase II inhibitor. The method is effective, resulting in good density of SV and no detectable SNV. The authors provide a full characterization of the mutants, their phenotypes, and their genomes. The 34-sample selected for genomic analysis is sufficient to make firm conclusions.

- The manuscript is clearly written and illustrated.

- The manuscript does a very good job at covering the phenotypic and molecular analysis for this type of mutagenesis. For example, they highlight the difference between short and long reads in the identification of SV.
- The figures are very clear, with the exception of Fig. 3, which I found harder to follow. It would be enhanced by describing the candidate lesion(s) in the first panel of each mutant series. This would clarify the expectation. For example, larger indels (not examined here) should be associated with higher (insertion) or lower (deletion) expression of the affected genes. In the cases presented in Fig.3, the structural changes do not suggest obvious hypotheses. The authors examine the regions near breakpoint of inversions or near small indels. It makes sense, but it does not make the figure very digestible. The connected text in the results, on the other hand, is very clear. Perhaps, making the conclusions in the figure legend as well? As a connected thought, it would have been useful to provide expression data for a large indel exemplifying the cis/trans nature of regulatory changes.

**Response:** Thank you for your helpful comments. The RNA-seq data is now presented before the DNA sequencing data to clarify the role of the RNA-seq data in this study. The previous Figure 3 is now Figure 2. We have clarified the presentation by combining original Fig. 3C and 2E into a single panel (Fig. 4F); moving 3J to Figure 4E; removing what was 3I; and moving the original 3B, 3E-G to Figure S9. In Figure S9, a label for the type of SV was added to the scatterplots of expression of genes surrounding the SVs. For further clarity, the panel describing the inversion in *BR-like dwarf* is also now plotted in the same way as those of *short-internode dwarf*. We agree it would be informative to provide expression data for a large indel to determine the extent of *cis* or *trans* effects, but we did not find any large indels in the samples we sequenced with long-reads.

We agree with the reviewer that some of the mutants we have generated would be great material for further studying cis/trans nature of regulatory changes. We are strongly interested in this question; it is certainly a subject for a future publication.

- Deletions and other rearrangements may affect meiosis as noted by the authors. In addition, they can display gametophytic phenotypes and a deficit in transmission. The likelihood increases with the size of the indel. Large indels are not transmitted. Accordingly, for indels above a certain size, it is not possible to determine the number of causal loci from F2 ratios.

**Response:** We agree that the impact of very large SVs on meiosis alters segregation ratios and prevents determination of causal loci from F2 ratios. Impacts on meiosis will also likely impact our ability to use techniques based on bulk segregant analysis to finely map causal mutations. It is important to note that such mutations comprise only a fraction of all detected SVs.

Identification of multiple loci causing a phenotype in plants carrying large SVs will therefore require other approaches. For example, structural variation callers can be used to identify the boundaries of SVs like duplications or deletions. RNA-Seq can be used to identify genes at the SV whose expression is highly effected by cis-regulatory changes. To test if those SVs are responsible for the phenotype, genes at SV boundaries along with the novel promoters can then be reintroduced as transgenes. This should enable one to identify combinations of mutated genes that are responsible for the phenotypes.

- Although the use of topo II inhibitors for mutagenesis in plants is novel, the mutagenic effects described here are well documented in animals. This should be acknowledged (e.g. Heisig, Mutagen. 2009; Ferguson, Env Mol Mutagen. 1994)

**Response:** We have acknowledged prior work demonstrating the mutagenic impact of etoposide using primary literature as well as more recent references. These include references # 43-45.

**Referees cross-commenting** 
I also found the expression analysis confusing and in need of revision. One reason is that a set of clear expectations were not provided. I believe that the RNAseq analysis is expected to help identify the gene(s) that underlie a trait. For example, genes located on an indel are likely to display expression proportional to copy number. Also, a new junction or translocation could influence expression of the gene next to the break point. The authors should make this clear in the figure and the text.

**Response:** Organization of manuscript’s results has been changed to reflect that mRNA-seq was conducted to learn more about the phenotypes, not to definitively identify causal genes/mutations.

Reviewer #2 (Significance (Required)):

- I appreciated the description of the method. It should be widely applicable. In arabidopsis, it requires sustained growth in the presence of the inhibitor. This could limit its applicability. For example, it may not be effective with pollen because exposure by a short soaking period may not be sufficient. Culturing of large seeded species is possible, but adds complexity. In this context, radiations have advantages. I do agree with the authors on the difficulty in identifying a source. However, once one is found, radiation treatment is very simple and convenient.

- The manuscript describes a useful tool and the connected spectrum of mutations. It has the novelty, quality, and relevance to represent a significant contribution to plant biology and to be of broad interest.

**Response:** Thank you for your feedback. For Arabidopsis, we germinated and grew seeds on media containing etoposide for about two weeks (see Methods). In work that is not yet ready for publication, we have taken the same approach with a legume species and other Brassicaceae that have substantially larger seeds. We find that we need to use a higher dose of etoposide to induce phenotypes, but that it is easy to germinate and grow large-seeded plants for a couple of weeks on media containing etoposide. We don’t anticipate that seed size will be limiting for this method.

We agree that this technique will not work for pollen; it will only work for tissues with significant levels of DNA replication. However, this technique alleviates the need for collecting pollen. For example, pollen irradiation has been used to create poplar with structural variants. However, if using etoposide-based mutagenesis, one could grow poplar seeds, cuttings, explants, embryos, or calli on etoposide-containing media.

**Reviewer 3**

Summary:

The manuscript entitled "A simple method to efficiently generate structural variation in plants" by Bechen et al. investigated an efficient mutagen for inducing large structural variations in plants, replacing traditional irradiation methods with a chemical mutagenesis strategy. The study examined the effects of etoposide, a DNA topoisomerase II inhibitor, on structural variations and demonstrated that etoposide treatment induces a wide range of phenotypic and genome changes, including inversions, duplications, and deletions. Additionally, the authors analyzed the relationship between gene expression changes and genomic alterations to identify potential causal genes underlying specific phenotypes. While their findings provide clear and reliable evidence of structural variations induced by etoposide, I have several suggestions to enhance the clarity of their results, as detailed below.

**Response:** We thank the reviewer for their feedback. It has helped improve the presentation and clarity of our results.

Major comments:

1. Lines 166-169: My understanding is that you selected etoposide-treated M1 plants based on specific phenotypes, and observed their M2 and M3 progeny, categorizing them as either phenotype-positive or phenotype-negative. In Table S3, phenotypes other than BR-like dwarf, virescent, and short internode dwarf are not mentioned. Does this indicate that these other lines did not exhibit heritable phenotypic traits? If other lines showed some phenotype changes, could you incorporate progeny relationships along with phenotype information into Table S3? Additionally, in Figures S2 and S3, you reference 26A lines. Did they exhibit similar phenotypic changes among them?

**Response:** Unfortunately, we do not have detailed phenotypes of each chosen M1 line. Most lines had one or more of the phenotypes we mention in the results – “Those exposed to 160 µM of etoposide exhibited significantly more abnormal phenotypes than DMSO only or 80 µM etoposide plants, including loss of apical dominance, gnarled leaves, reduced plant size, seed abortion, and lower seed number at maturity (Figure 1A).” It is important to note that M2 phenotypes were not observed in M1.

Table S3 is now Table S7. Only some mutants lines or lineages that were sequenced had one of the scored phenotypes in M2 (10B,13B, 1A, 1B, 21A, 24B, 26A, 34C, 5A,9A). Of these, we have formally tracked inheritance of only 13B, 1A, 34C, and 5A over multiple generations. We do not have similar data for other sequenced lines . We also sequenced some lines (17B, 21B, 26C) without any mutant phenotypes to assess if plants lacking visible phenotypes still carried SVs. Indeed, as described in Table S9, all three of these lines carried small SVs, which might not affect genes that create an obvious visible phenotype under our growth or observation conditions.

Yes. 26A siblings all exhibited the same flat leaf phenotype.

Overall, our data suggests that mutant phenotypes and their causal SVs can be stably transmitted through multiple rounds of meiosis.

2. Lines 187-189 and Figure S4: The assessment of repeat copy number variation provides valuable insights. However, based on the figure, the conclusion that "etoposide treatment likely did not trigger genomic instability in repetitive DNA" is difficult to interpret. Could you modify the figure into a box plot with raw data points and include a statistical analysis to support this conclusion?

**Response:** The figure has been modified to a box plot and is now presented as main Figure 3. Wilcox test has been performed and shows no significant difference in read depth over NOR2, NOR4, and telomere regions between control and etoposide-treated lines.

3. Line 200 and Figure S8A: You state that SNV analysis identified a similar number of SNVs in treated and control plants. However, this is not easily interpretable from the figure. Could you include a statistical comparison between etoposide-treated and control plants? For example, EMS mutagenesis is known to induce specific G/C → A/T transitions. Did etoposide-treated and control plants exhibit the same types of nucleotide changes, or were there differences in the mutation spectrum?

**Response**: This figure has been modified to assess the entire mutational spectrum of SNVs, including statistical comparisons, and is now part of Figure 3. We have also added the following text on line 244: “However, SNV analysis identified a comparable spectrum and number of SNVs in etoposide-treated and control lines (Figure 3), suggesting that etoposide did not induce excess SNVs.”

4. Lines 219-220: Your conclusion clearly demonstrates the detection of numerous structural variations using both short- and long-read sequencing technologies. Could you provide a summary table listing the detected mutation positions? Since short-read sequencing is generally less effective in detecting large structural variations, I am particularly interested in evaluating the accuracy of Lumpy Express in identifying mutations.

**Response:** Short-read sequencing and Lumpy Express are unsurprisingly less effective in detecting large structural variations when compared with long-read based approaches. SVs detected by Nanopore were missed by short-read sequencing and Lumpy Express. However, it is hard to benchmark the efficacy of Lumpy Express as only a few lines were sequenced by both Nanopore long-read sequencing and short-read sequencing. After removing SVs that were also present in control lines, we could identify only one SV detected by both Lumpy Express and Nanopore sequencing; this SV is a deletion. In plant 1A_4_5, which was sequenced by short reads, Lumpy Express called a 70 bp deletion at Chr 5: 5776579. SV calling using Nanopore-generated sequence of a sibling plant, 1A_4_11, identified a 70 bp deletion at Chr 5:5776578. The SVs identified by Lumpy Express are presented in Table S9. Those identified from long-read data are in Table S11.

5. Figures 3E-G: To facilitate a clearer comparison of the effects of structural variations on gene expression between BR-like dwarf and short internode dwarf, could you add an average trend line to the figures, similar to Figure 3B?

**Response:** An average trend line has been added to these plots. These data are now presented in Figure S9.

Minor comments:

Line 105 and Figure 1A: In the manuscript, etoposide concentrations are stated as 0, 40, 80, and 160 μM, whereas Figure 1A labels the concentrations as 0, 80, 160, and 320 μM. Should the figure be updated to 0, 40, 80, and 160 μM for consistency?

**Response:** Thank you for the comment. We updated the results section to make concentrations listed consistent with methods (0, 20, 40, 80,160, 320, and 640 µM) and added additional description of seedling growth such that all concentrations are described. We also updated references to the figure such that only sentences regarding concentrations with photos reference Fig 1A.

Figure 1B legend: Typographical error: "roundsof" → "rounds of".

**Response:** Corrected.

Line 109: Do you have a summary table for the M1 generation? If so, could you provide it as a supplementary table?

**Response:** We regretfully do not have the data to populate a summary table for the M1 generation. As described above, most M1 plants have one or more of the following phenotypes mentioned in text: “loss of apical dominance, gnarled leaves, reduced plant size, seed abortion, and lower seed number at maturity “

Line 119: Figure 1B only defines developmental stages. To improve clarity, consider revising "Figure 1B" to "Figure 1B-F", allowing readers to easily understand the corresponding figures.

**Response:** We updated the Figure reference to Fig. 1B-F.

Line 121: The citation "(Figure S1, Table S1)" would be clearer if placed at the end of the sentence.

**Response:** The citation was moved to the end of the sentence.

Lines 137, 148, 167: To maintain consistency with Figure 1C-F and the manuscript's logical flow, could you standardize the order of phenotypes as "virescent, short internode dwarf, and BR-like dwarf" instead of the current variation?

**Response:** We have standardized the order of the figures and the discussions of phenotypes in the text as: *BR-like dwarf, short-internode dwarf, virescent,* then *variegated.*

Line 139: Why is "Figure 1B" referenced at this position? Would it be more appropriate to remove this reference?

**Response**: Figure 1B shows that the phenotypes were able to be transmitted at least until the M5 generation, thus the reference.

Figure S7 legend: Typographical error: "to to" → "to".

**Response:** Corrected.

Figure S8B (Chromosome 5 labels): Could you adjust the position labels to maintain a consistent format with other chromosomes?

**Response:** This figure has been modified on request of another reviewer such that this is no longer applicable.

Lines 262, 277, 279: "Figure S11" should be corrected to "Figure S10".

**Response:** Corrected.

Line 269: "Figure S10" should be corrected to "Figure S11B-H".

**Response:** Corrected.

Reviewer #3 (Significance (Required)):

In mutation studies aimed at inducing large-scale genomic variations, irradiation has traditionally been the primary method for mutagenesis. However, this study proposes a more efficient and accessible alternative using chemical mutagenesis with a DNA topoisomerase II inhibitor. Genomic analysis of mutants generated through this treatment revealed extensive genomic alterations, with a mutation frequency exceeding that of gamma irradiation-induced mutants. These findings suggest that this approach has the potential to advance mutation research for plant biologists and breeders seeking efficient methods for trait improvement. Furthermore, the authors integrate RNA-seq analysis for selected traits, demonstrating a systematic workflow for candidate gene identification and facilitating the determination of causal genes.
