# Supplementary material for: A simple method to efficiently generate structural variation in plants
Source: PLoS Genet. 2025 Dec 18;21(12):e1011977. doi: 10.1371/journal.pgen.1011977 (PMC12725597; doi:10.1371/journal.pgen.1011977)
Supplement: Attachment — Submitted filename: Rebuttal.docx [file pgen.1011977.s024.docx]

Reviewer's Responses to Questions

**Comments to the Authors:
Please note here if the review is uploaded as an attachment.**

Reviewer #1: The revision satisfies my criticism.

We thank the reviewer for their previous suggestions.

Reviewer #2: I have to say that I was not involved in reviewing the first version of the paper. I read the revised manuscript as well as the rebuttal letter of the authors with interest. Using etoposide, the authors are able to induce a larger number of structural variations in Arabidopsis, mainly insertion and deletions, which they also characterize by long read sequencing. The authors were able to correlate SVs with specific phenotypes, however not all SVs are correlated with phenotypic changes. These are interesting data and I regard them interesting for a large audience. Nevertheless, I feel that the authors are a bit too positive in the discussion comparing their results with irradiation mutagenesis. Moreover, in the last years the induction of directed Mb-sized SVs especially inversions by CRISPR/Cas has been achieved in a row of plant species and I think that this fact should be mentioned in the discussion, too

We thank the reviewer for their appreciation of our study. To address the comments on discussion, we have made the following changes:

1. **Reviewer’s comment:** “Nevertheless, I feel that the authors are a bit too positive in the discussion comparing their results with irradiation mutagenesis. “

   **Our response:** We have changed the text in the manuscript (Lines 392-393) to read “*While the exact efficacy of irradiation or etoposide-based mutagenesis can vary by experiment, currently available data suggests that etoposide treatment exceeds irradiation by producing more than 2.5 events per mutagenized plant.* “ (newly added text is underlined” ).
2. **Reviewer’s comment:**  Moreover, in the last years the induction of directed Mb-sized SVs especially inversions by CRISPR/Cas has been achieved in a row of plant species and I think that this fact should be mentioned in the discussion, too

   **Our response:** Our protocol is designed for random mutagenesis, and for difficult to transform species. It seeks to complement CRISPR-programmable SVs. We have previously mentioned this in the introduction section- “…*information that can be used for targeted CRISPR-mediated restructuring of crop genomes.*” We have now added an additional line (Lines 455-458) to the discussion – “*Our method will generate mutant libraries that can be used directly for research or breeding, or provide targeting information for CRISPR-based tools that have recently created mega-base scale SVs in crop species.*”

Reviewer #3: This manuscript, “A simple method to efficiently generate structural variation in plants” by Bechen et al. presents an innovative approach to plant mutagenesis through chemical treatment with the topoisomerase II inhibitor etoposide. The authors show that, unlike commonly used chemical mutagens (e.g., EMS, sodium azide) that mainly induce SNPs, etoposide efficiently generates large structural variants (e.g., indels, inversions, duplications, and translocations) while maintaining wild-type-like SNV frequencies. This method represents a promising alternative to irradiation, offering a more accessible method for inducing complex mutations relevant for plant biology and breeding.
The study combines phenotypic characterization, whole-genome sequencing (both short and long reads), and transcriptome analysis to identify structural variants and potential causal mutations. The manuscript is clearly written, well-illustrated, and supported by thorough experimental work that strongly supports authors conclusions.
Overall, the manuscript introduces a novel, valuable and broadly applicable tool for generating genomic structural variation in plants, with strong potential to impact both fundamental research and applied breeding.

**Our response:** We thank the reviewer for their comments.

I only have a couple of minor comments on the data and interpretation:
- While the method shows promise, its applicability may be limited by the need for sustained exposure to the inhibitor, which may be less practical in certain contexts compared to radiation. My recollection is that etoposide is relatively unstable in solution and light sensitive. I therefore wonder whether etoposide remains active after several days (up to 2 weeks)?

**Our response:** It is plausible that irradiation might be more useful in certain contexts.

The reviewer is also correct that etoposide is not very stable. However, it’s stable enough for the two-three-week exposure that is prescribed here for mutagenesis. Seedling growth is stunted on etoposide relative to media with just solvent. This leads to a reasonable argument that seedling growth will remain slow as long as there is sufficient etoposide in the media. Our observations indicated that seedling growth rate did not increase immediately after two weeks of exposure, suggesting that there was enough etoposide in the media for the duration of the exposure. Furthermore, we found that many M1 lineages had mutations, suggesting that the current protocol works, at least in the case of Arabidopsis.

This could explain the varied efficiency of etoposide treatment reported by the authors.

We do not think the varied efficiency is linked to etoposide degradation in the media. We noticed that seven days after germination on different batches of etoposide, seeds grown on an older, previously-tested batch of etoposide showed more growth stunting than those grown on a newer batch. We have had this experience at least three times – once was with etoposide from the same company and twice was with etoposide from two different companies.

 Did the authors attempted repeated or renewed etoposide treatments after for example 1 week (e.g. by adding fresh etoposide in the medium if transferring to new plates is tedious)? Or by treating few days after germination? (Since treatment requires active DNA replication, applying it directly to seeds may not be optimal).

The scenario suggested by the reviewer is plausible. We mentioned in the paper that prolonged exposure to etoposide can increase the number of mutations per M1 plant. This recommendation is based on experiments where we grew seedlings on liquid MS media in which etoposide treatments were renewed each week. But, M1 lethality was increased upon transfer to soil after four weeks of this treatment. However, we have not observed M2 progeny from these plants and thus cannot comment on whether mutational frequency is further improved.

Minor point : Cytotoxic drugs usually require neutralization for safe disposal. However, there is no mention of such procedure in the method. Was any neutralization procedure applied that is worth mentioning?
**Our response:** We acknowledge the need for safe disposal but we did not find clear information on whether low or high pH can hydrolyze etoposide. We therefore sent all our etoposide waste to the incinerator. Exposure to etoposide in the lab was controlled by the use of personal protective equipment.
We have added the following lines to Text S1: “Safety***:*** *Etoposide is a cytotoxic drug and etoposide containing waste should be treated in accordance with institutional guidelines. In our case, all etoposide- containing waste was incinerated. Previous studies have shown that etoposide may be degraded by potassium permanganate or bleach (Benvenuto et al. 1993) but we have not tested this approach. All exposure to etoposide was controlled via the use of personal protective equipment.”*

- Line 214- 218 : The text gives impression that DSBs leads to SNV. However, my understanding is that while a DSB can theoretically be repaired in a way that leads to just a single-base substitution, this is not the most common outcome. Instead, DSB repair most often results in small insertions or deletions due to non-homologous end joining NHEJ and/or Microhomology-mediated end joining, or, as described by the authors in the manuscript, larger structural variants (SVs) such as inversions, duplications, or translocations.

**Our response:** Etoposide has a wide mutational spectrum. In the chicken DT40 lymphoblast cell line, etoposide exposure increased base substitution somewhat but did not seem to increase indel frequency (Szikriszt et al. 2016). In contrast, etoposide treatment in yeast, as measured through the mutation rates at the *CAN*1 locus, shows evidence of elevated duplications and 1bp insertions (Stantial et al. 2020). These results suggest that the mutagenic consequences of etoposide exposure may be species, and perhaps exposure-level, dependent. Observations of mutagenic consequences in older papers might also be impacted by technological limitations.
To take into account all possibilities, we have amended lines 215-222. They now read: “*The short-read data was also used to test if etoposide treatment resulted in increased single nucleotide variants (SNVs), or short indels . SNV and short indel analysis identified a comparable number and spectrum of SNVs in etoposide-treated and control lines (Figure 2C, D), suggesting that etoposide did not induce excess SNVs or short indels. Nevertheless, we cannot completely rule out the possibility that some SNVs and short indels may arise due to exposure to etoposide. Therefore efforts by users of etoposide mutagenesis to identify mutations underlying phenotypes of interest should also employ analyses that can accurately identify SNVs and small indels.*  “

All events observed and described by the authors are > 35 bp. Have the authors checked for small Indels (2-30 bp) typically associated with end-joining ? (Of note, cells lacking NHEJ factors are usually hypersensitive to etoposide).

**Our response:** We tested the hypothesis that etoposide treatment induces SNVs and small indels by examining the number and spectrum of single nucleotide variants and small indels in progeny of DMSO and etoposide treated lines. Analysis presented in Figure 3A shows that etoposide treatment did not elevate the frequency of SNVs and indels. We therefore did not pursue this line of analysis further. However, we cannot completely rule out that some SNVs and indels may arise as a result of etoposide treatment. We have therefore added lines 218-221 – “*Nevertheless, we cannot completely rule out the possibility that some SNVs and small indels may arise due to exposure to etoposide. Therefore, efforts to identify mutations must employ at least one sequencing technology that can accurately identify SNVs and small indels.* “

- Line 297-303 : Do the authors have any comments on the upregulation of meiotic genes in Virescent plant leaves? Are these plants later affected in fertility?

**Our response:** We did not notice any impact on fertility in the *virescent* mutants.

- Table S7-to S9 provide the complete RNAseq data. However, the authors might also consider including a list containing only the few deregulated genes, which would be more straightforward to interpret for the reader.

**Our response:** All the data from DESeq2 output is in the supplemental tables and can be easily sorted in excel.
