# Supplementary material for: A simple method to efficiently generate structural variation in plants
Source: PLoS Genet. 2025 Dec 18;21(12):e1011977. doi: 10.1371/journal.pgen.1011977 (PMC12725597; doi:10.1371/journal.pgen.1011977)
Supplement: Attachment — Submitted filename: Rebuttal 111325_2.docx [file pgen.1011977.s025.docx]

**Response to reviewers**

**Reviewer’s comment:** Moreover, in the last years the induction of directed Mb-sized SVs especially inversions by CRISPR/Cas has been achieved in a row of plant species and I think that this fact should be mentioned in the discussion, too\

**Our response:** As described previously, we had added an additional line (Lines 455-458) to the discussion – “*Our method will generate mutant libraries that can be used directly for research or breeding, or provide targeting information for CRISPR-based tools that have recently created mega-base scale SVs in crop species.*”

**Editor’s note in response:** Thank you very much for this revised version of your manuscript. It is almost ready for PLOS Genetics's publication but we would like to ask you to add details in your answer to Reviewer2/comment 2: please add the **relevant references** to support the added sentence: " Our method will generate mutant libraries that can be used directly for research or breeding, or provide targeting information for CRISPR-based tools that have recently created mega-base scale SVs in crop species **(REFS)**"

**Our response:** We apologize for out oversight. We have now added the following references to the manuscript:
74.	*Puchta H, Houben A. Plant chromosome engineering – past, present and future. New Phytologist. 2024;241: 541–552. doi:10.1111/nph.19414*

*75. Schwartz C, Lenderts B, Feigenbutz L, Barone P, Llaca V, Fengler K, et al. CRISPR–Cas9-mediated 75.5-Mb inversion in maize. Nat Plants. 2020;6: 1427–1431. doi:10.1038/s41477-020-00817-6*

*76.Schmidt C, Fransz P, Rönspies M, Dreissig S, Fuchs J, Heckmann S, et al. Changing local recombination patterns in Arabidopsis by CRISPR/Cas mediated chromosome engineering. Nat Commun. 2020;11: 4418. doi:10.1038/s41467-020-18277-z*

*77.Lu Y, Wang J, Chen B, Mo S, Lian L, Luo Y, et al. A donor-DNA-free CRISPR/Cas-based approach to gene knock-up in rice. Nat Plants. 2021;7: 1445–1452. doi:10.1038/s41477-021-01019-4*
